# Genetic Profiling Differentiates Second Primary Tumors from Metastases in Adult Metachronous Soft Tissue Sarcoma

**DOI:** 10.1155/2008/431019

**Published:** 2009-02-02

**Authors:** Josefin Fernebro, Ana Carneiro, Anders Rydholm, Henryk A. Domanski, Anna Karlsson, Åke Borg, Mef Nilbert

**Affiliations:** ^1^Department of Oncology, Institute of Clinical Sciences, Lund University Hospital, 221 85 Lund, Sweden; ^2^Department of Orthopedics, Institute of Clinical Sciences, Lund University Hospital, 221 85 Lund, Sweden; ^3^Department of Pathology, Institute of Clinical Sciences, Lund University Hospital, 221 85 Lund, Sweden; ^4^Clinical Research Centre, Hvidovre Hospital, Copenhagen University, 2650 Hvidovre, Denmark

## Abstract

*Purpose*. Patients with soft tissue sarcomas (STS) are at increased risk of second primary malignancies, including a second STS, but distinction between metastases and a second primary STS is difficult. *Patients and Methods*. Array-based comparative genomic hybridization (aCGH) was applied to 30 multiple STS of the extremities and the trunk wall from 13 patients. Different histotypes were present with malignant fibrous histiocytomas/undifferentiated pleomorphic sarcomas being the predominant subtype. *Results*. aCGH profiling revealed genetic complexity with multiple gains and losses in all tumors. In an unsupervised hierarchical cluster analysis, similar genomic profiles and close clustering between the first and subsequent STS were identified in 5 cases, suggesting metastatic disease, whereas the tumors from the remaining 8 patients did not cluster and showed only weak pairwise correlation, suggesting development of second primary STS. *Discussion*. The similarities and dissimilarities identified in the first and second STS suggest that genetic profiles can be used to distinguish soft tissue metastases from second primary STS. The demonstration of genetically different soft tissue sarcomas in the same patient suggests independent tumor origin and serves as a reminder to consider development of second primary STS, which has prognostic and therapeutic implications.

## 1. INTRODUCTION

The first observations of an increased risk of secondary sarcoma development came from individuals treated for childhood cancers such as retinoblastoma, leukemia, Wilms' tumor, Hodgkin's lymphoma, and sarcoma [1–13]. However, also adult soft tissue sarcoma (STS) patients have been shown to be at higher risk of a second primary malignancy, with a particularly increased risk of a second primary STS [14–17]. STS have been associated with several hereditary syndromes, the most common being neurofibromatosis and the Li-Fraumeni syndrome [18]. Treatment-induced sarcomas include sarcomas associated with Stewart-Treeves syndrome and radiation-induced angiosarcomas, which develop median 10 years after radiotherapy [19]. However, after exclusion of STS in neurofibromatosis patients and STS that developed in irradiated fields, an increased risk of a second primary STS remains [17]. Metachronous STS has been described in 1% of sarcoma patients [16, 17, 20] and this observation constitutes the basis for our study on similarities/differences in the genetic profiles of tumors from patients with multiple STS. We applied array-based comparative genomic hybridization (aCGH) that utilizes BAC clones with tiling coverage of the whole genome and allows detailed copy-number analysis, to a series of 30 metachronous STS of different histopathological subtypes from 13 patients.

## 2. PATIENTS AND METHODS

### 2.1. Patients

Adult patients (≥16 years of age) who developed two or more STS at different anatomical sites before development of any detectable pulmonary metastases were eligible for the study. Patients with neurofibromatosis type I and the Li-Fraumeni syndrome were excluded. In the southern Swedish cancer registry, 20 patients who fulfilled these criteria were identified. The tumors had been operated either at the musculoskeletal tumor center in Lund (*n* = 24) or at local hospitals in the southern Swedish health care region (*n* = 6). The clinicopathological reports were reviewed to confirm tumor location and to rule out that the second primary STS represented a local recurrence and the histopathological slides were reviewed by a sarcoma pathologist (H.D.) to confirm the diagnoses.

Frozen tumor tissue was available from 16 tumors and paraffin-embedded tissue was used from 28 tumors without systematic differences related to tumor source. After DNA extraction, 7 individuals (14 tumors) were excluded because of poor DNA quality in at least one of the tumors from the same patient. High-quality aCGH data were obtained from 30 tumors (in 15 of which DNA was extracted from frozen tissue) from 13 patients (Table 1). These patients contributed with two to four STS and were mean 73 (28–83) years at diagnosis of the first STS. The second STS developed median 3 (1–7) years after the first STS. No neoadjuvant chemotherapy was given, and only one patient (case 5) had recieved postoperative chemotherapy after the first STS. Radiotherapy had been administered to four patients (postoperatively in case 5, 6, and 8, and preoperatively in case 1), but none of the second STS developed within the irradiated field.

Clinical data for the 13 cases are presented in Table 1. The lower extremity was the most common tumor site (16 tumors) and 28 tumors were high-grade (grades 3 and 4 on a 4-tiered scale). The first STS included eight malignant fibrous histiocytomas/undifferentiated pleomorphic sarcomas (MFH/UPS), two leiomyosarcomas, two malignant peripheral nerve sheath tumors (MPNST), and one pleomorphic liposarcoma. The histopathological diagnosis of the second STS differed from the first in two patients; a leiomyosarcoma was diagnosed in a patient with two prior MFH/UPS and a leiomyosarcoma was diagnosed in a patient previously operated on for an MPNST. In the remaining patients, including the three cases from which three or four distinct tumors were analyzed, multiple STS of the same histopathological type were diagnosed.

In 9/13 patients the STS developed at different anatomical locations, for example, different extremities or extremity and trunk wall. Three patients developed metachronous STS in the same extremity but at different locations, for example, lower leg and thigh (cases 8, 12, and 13) and one patient (no. 7) developed two STS in the same extremity; a deep-seated leiomyosarcoma in the medial thigh and five years later a subcutaneous leiomyosarcoma in the lateral part of the thigh (Table 1). Clinical follow-up was complete for a minimum of 8 years for the survivors. During follow-up, two patients (cases 12 and 13) developed local recurrences, 1 and 10 years after primary surgery. Lung metastases developed in 5/13 patients, median 50 (range 15–51) months after diagnosis of the primary tumor. Apart from the metachronous STS, two patients (cases 4 and 7) developed adenocarcinomas of the breast and the colon, respectively. Ethical permission for the study was granted from the Lund University ethics committee.

### 2.2. DNA extraction and array-based comparative genomic hybridization

Genomic DNA from frozen (*n* = 15) and paraffin-embedded (*n* = 15) tumors was extracted using the Wizard Genomic DNA Purification kit (Promega, Madison, WI) and overnight proteinase-K digestion treatment followed by phenol-chloroform purification. When paraffin-embedded tissue was used, a fresh 4-*μ*m section was obtained, stained with hematoxylin & eosin and a representative tumor area was chosen. Thereafter, 1-mm tissue cores were obtained and used for DNA extraction. The tissue cores were pretreated in xylene before proteinase-K treatment and phenol-chloroform purification. DNA quality was checked using a Ready-To-Go RAPD analysis kit (Amersham Biosciences, Buckinghamshire, UK), and the concentration was measured using a Nano drop (NanoDrop Technologies, Wilmington, Del, USA). Commercial genomic male DNA, derived from a pool of healthy individuals, was used as a reference (Promega, Madison, Wiss, USA). CyDye coupling/labeling was carried out using a random labeling kit (Invitrogen Life Technologies, Carlsbad, Calif, USA) according to the manufacturer's recommendations. In short, 2-*μ*g genomic tumor DNA and reference DNA were differentially labeled with fluorescent dyes, Cy3 for tumor tissue, and Cy5 for reference DNA. After a purification step, these were pooled, mixed with COT-1 DNA to block repetitive DNA sequences, dehydrated and resuspended in a formamide-based buffer (Invitrogen). The labeled DNA was then applied to arrays pretreated in washing solutions (Pronto! Microarray Reagent System, Corning Labsystems, Corning, NY, USA) and hybridization was performed for 48–72 hours at 37°C. The incubation was performed under cover slips for the DNA isolated from the frozen tumor material whereas the MAUI hybridization System (BioMicro systems Inc., Salt Lake City, Utah, USA) was used for the DNA derived from paraffin-embedded tumors. Dye-swaps (i.e., complementary hybridization in which Cy5 was used for tumor tissue and Cy3 for reference DNA) were used in three cases and allowed subtraction of dye-related noise. The slides were treated in post-hybridization washing solutions and finally scanned using an Agilent Microarray scanner (Agilent Technologies, Palo Alto, Calif, USA).

### 2.3. BAC array platform

The BAC array slides used were produced at the Swegene DNA Microarray Resource Center, Department of Oncology, Lund University. These have an average resolution of 80 kb and contain a total of 32 433 BAC clones from the 32 k human genome high-resolution BAC rearrayed clone set, version 1.0 from the BACPAC Resource Center at Children's Hospital Oakland Research Institute (Oakland, Calif, USA) (http://bacpac.chori.org/). The clones provide >99% coverage of the fingerprint map and current sequence assembly with a resolution of 100 kb.

### 2.4. Data analysis

Image analysis and data extraction were carried out using GenePix Pro 4.1.1.4 version (Axon Instruments Inc., Foster City, CA, USA) and the quantified data matrix was then uploaded into the web-based BioArray Software Environment (BASE; http://gothmog.thep.lu.se/int/index) [21], where all data management and analysis were carried out. The background correction and intensities of Cy3 and Cy5 were calculated using the median feature and median-local background intensities of the uploaded files, and the intensity ratios were calculated using the background corrected spot intensities by calculating the log_2_ ratios of tumor to reference intensity. In BASE a preliminary filter, based on the flagging in the image analysis, was applied, and spots with a diameter <55 *μ*m and a signal to noise (SNR) ratio ≤3 in the tumor or reference channel were flagged as “bad” and filtered away from further analysis. The intensity-dependent LOWESS algorithm [22] was used to normalize the data within individual arrays. To correct for spatial bias, the data were normalized within groups of 8 print-tip blocks. A moving average smoothing algorithm with a 250 kbp sliding window was then applied, and a BASE-adapted CGH-plotter software was used to identify regions of gains and losses [23]. In the CGH-plotter, each clone was assigned a calculated level log_2_ ratio value, corresponding to the level that the clone belongs to, in order to reduce the noise. Hereafter an unsupervised hierarchical cluster analysis, using the Pearson correlation distance metric and the average linkage method, was applied to the data derived from the CGH-plotter (the TMeV application from the TM_4_ microarray software suite was used; http://www.tm4.org/mev.html). The CGH-plotter was also used to generate a ternary scale, where all clones were designated gained, lost or unchanged. These values were used to calculate the percentage of altered clones in each assay and the mean number of altered clones in tumor subgroups. Pearson correlation was used to determine the correlation between tumors within the same individual, based on the number of altered clones. Gains and losses were defined as a log_2_ ratio ±0.2. Amplifications were defined as clones with a log_2_ ratio ≥0.5, whereas high-level amplifications were defined as a log_2_ ratio ≥1.5. Homozygous deletions were suspected when the log_2_ ratio was ≤1.5.

## 3. RESULTS

Genomic profiles from the 30 metachronous STS of five different histopathological subtypes revealed multiple gains and losses and identified several high-level amplifications (HLA) and homozygous deletions (Table 2). The alterations affected mean 39 (9–70)% of the whole genome with 19% amplifications and 20% deletions. When the first STS (*n* = 13) were compared to the subsequent STS (*n* = 17) a small difference in the total number of alterations was found with 35 (16–54)% and, 42 (9–70)% of the genome altered, respectively. Several recurrent aberrations were identified with the most frequent changes (present in >60% of the tumors) being deletions of 10q24.3–25.2, 13q12.1–12.2, 13q21.1–21.2, 16q13–23.2, 18q12.2–12.3, and amplifications of 1q21.3–23.1 and 19p13.3.

**Figure 1:**
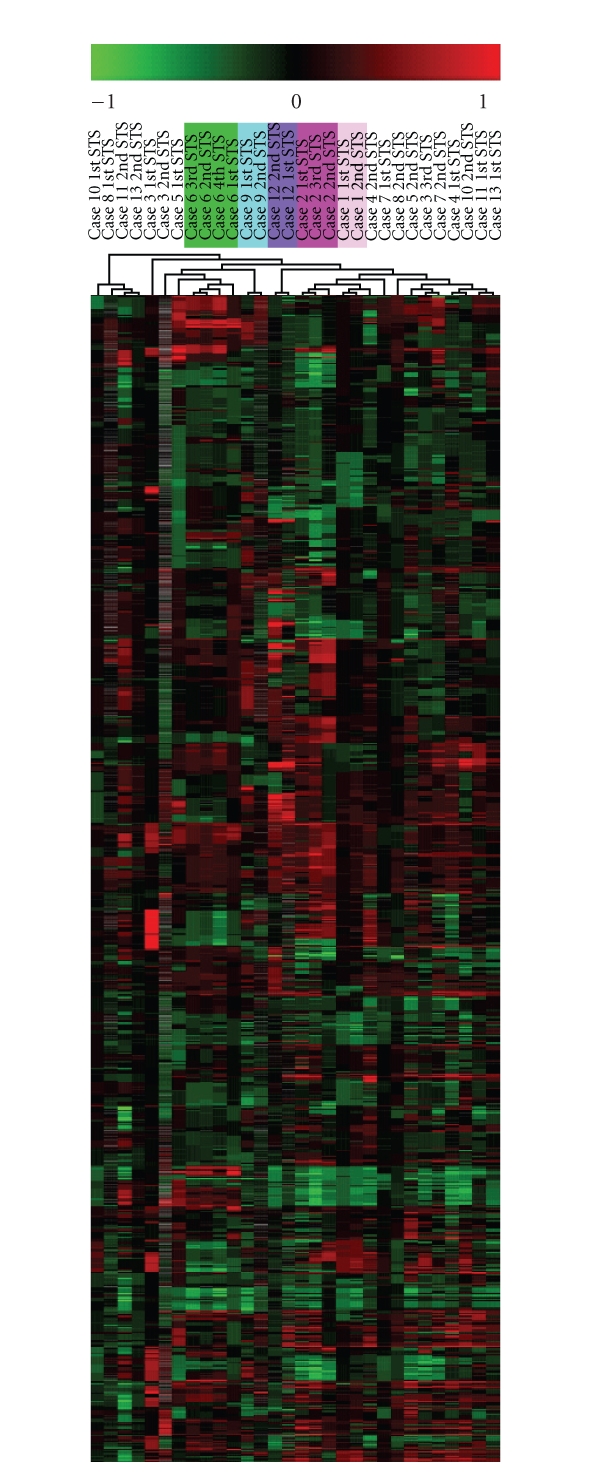
Unsupervised hierarchical cluster analysis of all 30 soft tissue sarcomas from 13 patients. The analysis was based on the ~17 000 clones that survived the preprocessing filters, and close clustering of the metachronous STS was demonstrated in 5 patients (case 1, 2, 6, 9, and 12 marked with different colours), whereas the STS from the remaining 8 patients were scatter in the analysis. Clustering was done using the TMeV application from the TM_4_ microarray software suit.

**Figure 2:**
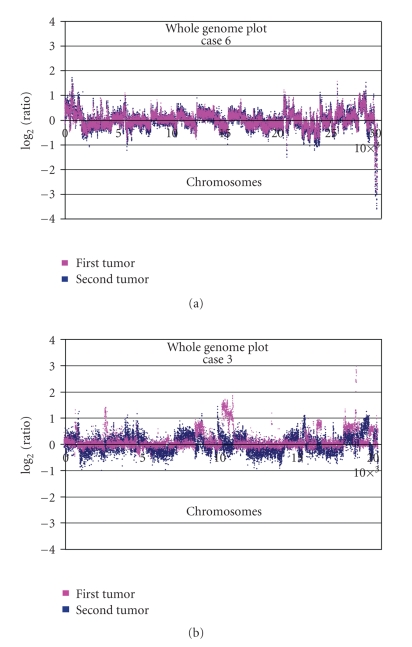
Superposition of whole genome plots from two different tumors from the same patient showing (a) similarities in gene copy number changes in two liposarcomas (case 6). (b) Differences in gene copy number changes in two malignant fibrous histiocytomas (case 3).

Unsupervised hierarchical cluster analysis, based on the ~27 000 clones that survived the filters in BASE, revealed close clustering of the tumors from five individuals without significant differences between the first and subsequent STS (43% and 41% of the genome altered) (Figure 1 and Table 1, cases 1, 2, 6, 9, and 12). These tumor pairs showed strong similarities between the genomic plots (Figure 2(a)) with a mean correlation of 0.7 (0.5–0.9). The many shared alterations outnumbered the few differences in all five cases and deletions identified in the first tumor were always present in the second STS. The median time interval between the first and second STS in these five patients was 1 (1–7) year, and two of these patients subsequently developed lung metastases. In the remaining eight cases STS from the same individual did not cluster together and showed a significantly weaker correlation, mean 0.1 (0–0.4). These tumors had pronounced intertumor variability (30% of the genome being altered in the first tumor compared to 42% in the subsequent tumors), which was comparable to the interpatient variability, which had a mean correlation of 0.1 (0.04–0.2). In four of these cases, deletions present in the first tumor were not present in the subsequent tumor, which supports independent tumor origin. The second STS in these eight cases developed median 4 (1–5) years after the first STS and three of the patients later developed pulmonary metastases.

## 4. DISCUSSION

Despite multidisciplinary and multimodality treatment, distant metastases develop in about 30% of STS patients. Hematogenous, pulmonary metastases predominate, whereas lymphatic spread occurs in <5% of the patients [19, 24]. Soft tissue metastases are rare and have mainly been reported in liposarcomas [16, 25]. Development of synchronous or metachronous STS has been described in several case studies, but it remains a rare clinical presentation and the interpretations hereof have varied [25–29]. Epidemiological data support an increased risk of a secondary primary sarcoma among adult STS patients [14, 17]. In order to reduce bias from inclusion of familial sarcoma syndromes, we excluded multiple sarcoma patients diagnosed with neurofibromatosis or the Li-Fraumeni syndrome. The only patient who developed more than one MPNST was carefully examined without any sign of neurofibromatosis (until death 3 years later). Since only one patient had received adjuvant chemotherapy after the first STS and none of the second STS developed in irradiated fields, the second STS studied are unlikely to represent treatment-induced secondary tumors.

Application of CGH in STS has mainly involved leiomyosarcoma and MFH/UPS and has in these highly malignant and pleomorphic STS subtypes demonstrated extensive genomic complexity with recurrent copy number changes, including losses of 2p, 2q, 10q, 11q, and 13q and gains of 1q, 5p, 8q, and 17p [30–36]. Several of these recurrent changes were also identified among the 30 STS in this study with the most frequent being deletions of 10q24.3–25.2, 13q12.1–12.2, 13q21.1–21.2, 16q13–23.2, 18q12.2–12.3, and amplifications of 1q21.3–23.1 and 19p13.3. In order to obtain as many tumor pairs as possible, frozen as well as paraffin-embedded tumor tissue was used. Among the tumors from which high-quality DNA was obtained, no differences were identified related to tumor source (Table 1). When the genomic profiles from the different tumor pairs were compared, five pairs showed highly correlated genomic profiles suggestive of metastatic disease, whereas eight cases showed different profiles suggestive of distinct primary STS. In the latter STS, the differences by far outnumbered the similarities, which resulted in a weak correlation, which was comparable to the interpatient variability. Importantly, there was no systematic difference in sample preparation methodology between the two groups suggestive of metastatic disease and distinct primary STS, respectively. The similarities and differences were evident in an unsupervised hierarchical cluster analysis (Figure 1) in which the five former tumor pairs clustered closely, whereas the latter eight did not.

Previous reports of multiple STS have predominantly involved liposarcomas. In a mixed series of nine STS patients who developed synchronous and metachronous STS, Grobemyer et al. identified liposarcomas (*n* = 5) and gastrointestinal stromal tumors (*n* = 5) as the most frequent subtypes whereas Blair et al. reported 16 patients with multiple STS of whom nine had liposarcomas [20, 37]. Our study only included successfully analyzed liposarcomas from one patient (case no. 6) who developed four liposarcomas in four years without signs of lung metastases and died from locally advanced tumor masses 22 months later. Antonescu et al. applied Southern blot analysis to tumors from six patients with multifocal myxoid liposarcomas and hereby verified monoclonality, thus demonstrating that multiple myxoid liposarcomas in the same individual most likely represent recurrent disease [16]. Similar genetic profiles were in our series present in metachronous STS of the same histopathological types; three MFH/UPS, one liposarcoma, and one leiomyosarcoma, which developed with median 1 year interval. Subsequent development of lung metastases occurred in two of these five patients (Table 1). Previous studies that have examined genetic differences between primary and recurrent STS have been carried out using conventional CGH and have demonstrated increasing genetic complexity from primary STS to a local recurrence [38–40]. However, the issue of development of second primary STS has to our knowledge not been addressed using genetic profiling. We demonstrate strikingly similar genetic profiles in the five STS likely representing soft tissue metastases with mean 43% and 41% of the genome altered and several shared deletions identified. This stands in contrast to the eight STS patients from which the metachronous STS showed different genetic profiles. Among these, multiple histologic subtypes (i.e., MFH/UPS, MPNST, and leiomyosarcomas) were present and four of the deletions identified in the primary tumors were not found in the second STS. In summary, the clinical presentation, histopathology, and the genetic profile support independent sarcoma origin in 8 of the 13 patients (Table 1). Although development of metachronous STS is rare, our demonstration of different genetic profiles in the majority of these cases serves as a reminder to consider independent tumor origin, which has implications for the choice of therapy, for example, use of adjuvant chemo- and/or radiotherapy after surgery for a second STS that should not per se be perceived to represent metastatic disease.

## Figures and Tables

**Table 1:** Summary of clinical data from the 30 STS analyzed from 13 patients.

Case no.	Sex	Age	Site	Type	Years after first tumor	Site	Type	Years after first tumor	Site	Type	Follow-up status/months	Tumor source frozen/paraffin
		*First tumor*			*Second tumor*			*Third tumor*				

*Similar genomic profiles suggestive of metastatic disease*												

1	M	80	Trunk wall	LMS	3	Left cheek	LMS				MET/50	f/f
2	F	82	Right upper arm	MFH/UPS	2	Left buttock	MFH/UPS	3	Scalp	MFH/UPS	MET/47	p/f/p
6	F	80	Right thigh	LS	1	Left shoulder	LS	2	Right lower leg^†^	LS	TD/22	f/p/p
9	F	79	Lower leg	MFH/UPS	1	Right thigh	MFH/UPS				NED/36	p/f
12	M	76	Right lower leg	MFH/UPS	7	Right thigh*	MFH/UPS				TD/164	p/f

*Different genomic profiles suggestive of second primary tumors*												

3	F	77	Right shoulder	MFH/UPS	5	Left thigh	MFH/UPS	9	Right lower arm	LMS	NED/163	f/f
4	F	75	Left lower arm	MFH/UPS	4	Right lower leg	MFH/UPS				NED/54	f/p
5	F	60	Right lower arm	MPNST	4	Right thigh	LMS				NED/56	f/p
7	M	83	Left thigh (laterally)	LMS	4	Left thigh (medially)	LMS				TD/28	p/f
8	M	73	Left knee	MFH/UPS	1	Left lower leg	MFH/UPS				MET/15	f/p
10	M	28	Right trunk wall	MPNST	1	Scalp	MPNST				TD/32	f/p
11	F	77	Right thigh	MFH/UPS	2	Back	MFH/UPS				MET/51	p/f
13	M	76	Right foot	MFH/UPS	4	Right thigh	MFH/UPS				MET/51	p/f

MFH/UPS = malignant fibrous histiocytoma/undifferentiated pleomorphic sarcoma, LMS = leiomyosarcoma, LS = liposarcoma, MPNST = malignant peripheral nerve sheath tumor. *Located 15 cm from the first tumor. ^†^The patient developed one additional LS, left thoracic wall. MET: metastases, TD: tumor death, NED: no evidence of disease.

**Table 2:** Recurrent high-level amplifications and homozygous deletions.

Chromosome	No of tumors	Cytoband	Start position	Size (Mbp)	HLA/Hz del*	Cancer related genes
1	2	1p32.1	58571482	1.2	HLA	*JUN*
1	3	1q44	241558190	1.0	Hz del	
3	2	3q23	141082484	0.51	Hz del	
3	3	3p12.1–3	78780218	7.0	HLA	
4	2	4q12	54016334	1.5	HLA	*PDGFRA, KIT, CHIC2*
6	3	6p 12.3–21.2	37360640	11.4	HLA	
9	6	9p21.3	21647433	2.4	Hz del	*CDKN2A, CDKN2B, MTAP*
10	2	10q23.31	89538420	1.5	Hz del	*PTEN*
11	2	11q13.4–5	71730097	5.3	HLA	
13	13	13q14.2	47901160	0.65	Hz del	*RB1*

*HLA = high level amplification, Hz del = homozygous deletion.
